# Measurement of arterial occlusion pressure using straight and curved blood flow restriction cuffs

**DOI:** 10.14814/phy2.16119

**Published:** 2024-06-19

**Authors:** Pat R. Vehrs, Ron Hager, Nathan Dayne Richards, Shay Richards, Luke Baker, Tyler Burbank, Shelby Clegg, Isabelle Katherine Frazier, Josh Richard Nielsen, Jessica Harkleroad Watkin

**Affiliations:** ^1^ Department of Exercise Sciences, 106 SFH Brigham Young University Provo Utah USA; ^2^ College of Osteopathic Medicine Rocky Vista University Ivins Utah USA; ^3^ Department of Statistics Ohio State University Columbus Ohio USA

**Keywords:** blood flow restriction, blood flow restriction exercise, blood flow restriction therapy, KAATSU

## Abstract

Arterial occlusion pressure (AOP) is influenced by the characteristics of the cuff used to measure AOP. Doppler ultrasound was used to measure AOP of the brachial and superficial femoral arteries using straight and curved blood flow restriction cuffs in 21 males and 21 females. Vessel diameter and blood flow were evaluated as independent predictors of AOP. Overall, there were no significant differences in AOP when using the straight and curved cuffs in the brachial (129 mmHg vs. 128 mmHg) or superficial femoral artery (202 mmHg vs. 200 mmHg), respectively. Overall, AOP was greater (*p* < 0.05) in males than in females in the arm (135 mmHg, 123 mmHg) and leg (211 mmHg, 191 mmHg). Brachial (0.376 mm, 0.323 mm) and superficial femoral (0.547 mm, 0.486 mm) arteries were larger (*p* = 0.016) in males than in females, respectively. Systolic blood pressure (SBP) and arm circumference were predictive of brachial artery AOP, whereas SBP, diastolic blood pressure, thigh circumference, and vessel diameter were predictive of superficial femoral artery AOP. Straight and curved cuffs are efficacious in the measurement of AOP in the arm and leg. Differences in vessel size may contribute to sex differences in AOP but this requires further investigation.

## 1 INTRODUCTION

Use of blood flow restriction (BFR) during low‐load strength training, aerobic training, neuromuscular stimulation, or passively is effective in stimulating muscle development or cardiovascular fitness (Cognetti et al., 2022). Low‐load strength training with BFR reduces the strain placed on the joints without compromising the benefits of resistance training (Cognetti et al., 2022). Training with BFR is a viable option during musculoskeletal rehabilitation following an injury or surgery as well as for others who are unable (or not willing) to train with heavy loads (Kim et al., 2017; Mattocks et al., 2018; Scott et al., 2015). Blood flow restriction is accomplished during training by placing a pneumatic cuff around the upper region of the arm or leg, and then inflating the cuff to a predetermined pressure that will partially restrict arterial blood flow and occlude venous blood flow (Iida et al., 2005, 2007; Loenneke & Pujol, 2009; Patterson et al., 2019; Pope et al., 2013; Scott et al., 2015). The current recommendation is to use an individualized cuff pressure representing 40%–80% of the limb's arterial occlusion pressure (AOP) (Cognetti et al., 2022; Hughes et al., 2017; McEwen et al., 2019; Patterson et al., 2019; Scott et al., 2015) during BFR training. Hence, the safe and effective use of BFR training depends, in part, on the ability to accurately measure each limb's AOP.

The AOP is defined as the lowest cuff pressure required, at a specific time, using a specific blood flow restriction cuff applied to a specific limb, at a specific location and body position, to occlude arterial blood flow distal to the cuff (McEwen et al., 2019). Intrinsic factors that contribute to AOP include limb dominance, circumference, and volume (Hughes et al., 2017; Mattocks et al., 2018; McEwen et al., 2019; Scott et al., 2015; Tafuna'i et al., 2021; Vehrs et al., 2024; Vehrs, Blazzard, et al., 2023; Vehrs, Richards, et al., 2023); systolic, diastolic, and mean arterial blood pressures (Hunt et al., 2016; Loenneke et al., 2015; Montoye et al., 2023; Vehrs, Richards, et al., 2023); and sex (Tafuna'i et al., 2021; Vehrs, Blazzard, et al., 2023). Extrinsic factors that contribute to AOP include cuff size (width), bladder design, material, and cuff position (Citherlet et al., 2022; Jessee et al., 2016; Loenneke et al., 2012; Montoye et al., 2023; Rolnick et al., 2023; Spitz et al., 2019, 2020).

The interest in the benefits of BFR has led to the availability of a variety of cuffs in the marketplace (Rolnick et al., 2023). The characteristics of the cuff (i.e., width, length, material, design of the bladder, and shape) collectively contribute to the degree of arterial occlusion achieved in the limb. For example, compared to narrower cuffs, wider cuffs compress a larger mass of tissue surrounding the blood vessel and yield a lower AOP (Jessee et al., 2016; Loenneke et al., 2012; Montoye et al., 2023). Although most blood flow restriction cuffs are “straight,” “curved” cuffs are also available (https://hpluscuff.com/; https://hokansonvascular.com/). Straight cuffs are designed to fit optimally on cylindrically shaped limbs (Pedowitz et al., 1993). Curved cuffs have a conical shape (longer at the top and shorter at the bottom) that provides a more secure fit around the tapered shape of the upper arm and upper leg (Pedowitz et al., 1993; Rolnick et al., 2023). Because the straight and curved cuffs are made of the same material, are the same size, and have the same bladder design, it could be presumed that the AOP values achieved with the two shapes of cuff would be similar. It could also be argued that when measuring AOP on a tapered arm or leg, a straight cuff may apply asymmetric pressures to the tissue beneath the cuff, thereby resulting in a higher AOP (Rolnick et al., 2023). Little research is available comparing AOPs measured when using straight and curved blood flow restriction cuffs. A 1993 study measured AOP on the upper arm and upper leg of 26 subjects using straight and curved cuffs (Pedowitz et al., 1993). Compared to when using an 8 cm wide straight cuff, using a curved cuff of the same size resulted in an AOP that was 25.4 and 4.3 mmHg lower in the thigh and arm, respectively (Pedowitz et al., 1993). Because straight and curved cuffs are readily available for the blood flow restriction practitioner and enthusiast, further research comparing the AOP values achieved with both shapes of cuffs is warranted (Rolnick et al., 2023).

Relatively few studies have compared limb AOP between males and females. For example, Jessee et al. (2016) reported small, yet statistically significant sex differences in brachial artery AOP (4–7 mmHg) that were of little practical importance. Greater sex differences in brachial artery AOP (≈12–15 mmHg) were reported by Mouser et al. (2017). Both large and significant (Tafuna'i et al., 2021) and nonsignificant (Vehrs, Blazzard, et al., 2023) sex differences in superficial femoral artery AOP have been reported. Because AOP is influenced by limb circumference (Hughes et al., 2017; Mattocks et al., 2018; McEwen et al., 2019; Scott et al., 2015; Tafuna'i et al., 2021), the differences in AOP between males and females could be presumed to be related to differences in limb circumference. To the contrary, the large sex differences in AOP reported by Tafuna'i et al. (2021) occurred despite no differences in limb circumference between the males and females. In addition, at any given limb circumference, there is a wide range of AOP values (Tafuna'i et al., 2021; Vehrs, Blazzard, et al., 2023) which might explain differences in AOP between individuals despite similar limb circumferences. Mounting evidence suggests that other factors contribute to differences in AOP between individuals and between males and females. Further exploration of sex differences in limb AOP may illuminate other factors that contribute to AOP. For example, although Mouser et al. (2017) reported sex difference in brachial artery blood flow and AOP, they did not report vessel size and did not evaluate the influence of blood flow on AOP. Because of the likely differences in blood flow and size of the brachial artery and the superficial femoral artery, evaluation of how these factors contribute to the AOP of the upper arm and the larger upper leg could help explain differences in AOP between individuals and between males and females.

The primary purpose of this study was to compare the AOP of the brachial artery and the larger superficial femoral artery of young healthy males and females when using straight and curved cuffs of the same size. A secondary purpose was to evaluate sex differences in AOP and variables that contributed to AOP.

## 2 MATERIALS AND METHODS

We measured the AOP of the brachial and superficial femoral arteries in the dominant limbs of young, healthy adult males and females when using a straight and curved blood flow restriction cuff (https://hpluscuff.com/). We also measured resting blood flow and brachial artery and superficial femoral artery vessel size (diameter), resting blood pressure, limb circumference and limb volume as potential variables predictive of AOP. This study was reviewed and approved by the Institutional Review Board (IRB2023‐256) prior to the collection of any data.

### 2.1 Participants

A total of 42 participants (21 males; 21 females) participated in this study. Exclusion criteria included obesity (BMI > 30 kg/m^2^) and self‐reported known risk factors for cardiovascular disease; one or more risk factors for thromboembolism, a diagnosis of or being treated for cardiovascular disease, renal disease, diabetes, or hypertension; or currently pregnant or less than 6 months postpartum (Jessee et al., 2016; Mattocks et al., 2017; Sieljacks et al., 2018). Risk factors for thromboembolism included a recent major surgery, pelvic, hip or long bone fractures, varicose veins, family history of deep vein thrombosis or pulmonary embolism, past events with venous thromboembolism, pregnancy, and use of oral birth control (Jessee et al., 2016; Mattocks et al., 2017; Sieljacks et al., 2018). Female participants completed their participation in this study at any point during their menstrual cycle (Stanhewicz & Wong, 2020). Participants were instructed to come to the lab in the fasted state (>4 h), having abstained from exercise and alcohol for 24 h and from food or drinks containing caffeine for at least 8 h (Crossley et al., 2019; Mattocks et al., 2017; Sieljacks et al., 2018). After the methods, expectations, risks, and benefits of the study were explained, each participant voluntarily provided written informed consent.

### 2.2 Anthropometric measurements

The height (cm) of each participant was measured using a calibrated wall‐mounted stadiometer scale (SECA Model 264; SECA, Chino, CA, USA). The body mass of each participant was measured using a digital scale (Ohaus Model CD‐33; Ohaus Corporation, Pine Brook, NJ, USA). The measured height and body mass was used to calculate body mass index (BMI; kg/m^2^). Limb circumference measurements were performed on the dominant arm and dominant leg, which was determined by asking the question, “If you were to throw (or kick) a ball, which arm (or leg) would you use?” (van Melick et al., 2017). We used three measurements to estimate the volume of the upper arm and upper leg. On the upper arm, we measured circumferences at the crease at the junction of the anterior border of the deltoid and the biceps brachii and at the proximal tip of the olecranon process of the ulna. These measurements were taken with the arm extended to the side and parallel to the floor with the elbow at 0°. On the leg, we measured the circumference of the thigh at the level of the gluteal fold and just above the proximal border of the patella. Thigh circumferences were measured with the participant in the standing position while bearing weight on the opposite leg. In order to calculate limb volume, we also measured the distance between the two landmarks where circumferences were measured on each limb. Circumferences were measured using a spring‐loaded Gullick measuring tape to reduce measurement error. The volume (m^3^) of the upper arm and upper thigh was calculated using the formula describing the volume of a truncated cone (Katch et al., 1973; Katch & Katch, 1974). A Lange skinfold caliper (Santa Cruz, CA) was used to measure the thickness of vertical skinfolds on the anterior midline of the thigh and the upper arm at the location where the blood flow restriction cuff would be placed to measure AOP. Circumferences and skinfold thicknesses were measured in triplicate. If two measurements were the same, that value was recorded; otherwise, an average of all three measurements was used.

After sitting in a semi‐reclined position with the legs extended (0° knee extension) and supported for 5 min, resting heart rate (HR), systolic (SBP) and diastolic (DBP) pressures were measured using an automated device (OMRON BP7200; OMRON Healthcare, Inc.). The average of two measurements was recorded and mean arterial pressure (MAP) was calculated from SBP and DBP.

### 2.3 Measurement of arterial blood flow

We used Doppler ultrasound (8 MHz, 55 mm Doppler ultrasound probe) and a LOGIQe ultrasound machine (GE Healthcare) to detect blood flow during the measurement of AOP and to capture a 30 s video of the brachial artery and superficial femoral artery for later analysis. Ultrasound gel was used as a medium between the ultrasound probe and the subject's skin. In order to evaluate the influence of vessel size and blood flow on AOP, we captured videos at locations where the blood flow restriction cuffs would later be placed to measure AOP. Thus, vessel size and blood flow were measured at locations that would be considered “beneath the cuff.” Using displayed pulse waves as reference points during the analysis of the saved video, the sonographer measured vessel diameter perpendicular to the direction of blood flow during systole. Time‐averaged velocity (cm/s) of blood flow over the 30 s video was recorded. Blood flow (mL/min) was automatically calculated by the ultrasound machine. The sonographer repeated the measurements during diastole. The measurements taken during systole and diastole were averaged to calculate blood flow values for data analysis.

### 2.4 Measurement of arterial occlusion pressure

After capturing videos for the analysis of blood flow, an inflatable straight or curved pneumatic blood flow restriction cuff was placed on the upper arm or the upper thigh. The two cuffs were inelastic, made of identical material (nylon), and were the same width (4 in, 10 cm). The length of the cuff to be used to measure AOP was based on the circumference of the participant's upper arm or upper thigh. The cuff was placed on the upper arm or upper thigh so that the center of the cuff bladder was positioned over the brachial artery or superficial femoral artery where 30 s videos were previously captured (Pickering et al., 2005; Spitz et al., 2020). Curved cuffs are conical in shape which theoretically fit more securely around the tapered shape of the upper arm and upper leg. The curved cuff was placed on the upper arm or upper leg so that the longer edge was placed on the top (superiorly) and the shorter edge was on the bottom (distally).

To detect blood flow and pulse waves, the ultrasound probe was placed distal to the cuff on the radial artery below the elbow or near the inferior border of the cuff on the medial side of the thigh when measuring AOP of the brachial and superficial femoral artery, respectively. The blood flow restriction cuff was inflated using a hand‐held sphygmomanometer (Model DS66; Welch Allyn, the Netherlands) with a gauge ranging from 0 to 300 mmHg with a ±3 mmHg certified accuracy. The cuff was inflated using a continuous protocol which involved initially inflating the cuff to 50 mmHg, then gradually increasing cuff pressure at a rate of approximately 10 mmHg/10 s. The AOP was defined as the lowest pressure at which both color blood flow and pulse waves were no longer visible on the ultrasound display. After AOP was recorded, the cuff was completely deflated and removed. Subsequent measurements of AOP were separated by 5 min (Jessee et al., 2016; Loenneke et al., 2012; Sieljacks et al., 2018). The order of AOP measurements (arm or leg, straight or curved cuffs) were randomized. So as to not bias measurements of AOP, the investigators monitoring blood flow using the ultrasound were blind to the pressure displayed on the pressure gauge of the sphygmomanometer.

### 2.5 Data analysis

The statistical analyses of data were performed using Statistical Analysis System version 9.4 (SAS Inc, Cary, NC, USA). Independent *t*‐tests were used to evaluate sex differences in age, height; body mass; BMI; resting SBP, DBP, and MAP; limb circumference; limb skinfold thickness; limb volume; vessel diameter; and blood flow. Because multiple comparisons were performed, a Bonferroni correction was used to adjust the *p*‐value to protect a family alpha level of 0.05. The primary variable of interest in this study was AOP measured using straight and curved blood flow restriction cuffs. To appropriately account for multiple sources of variability, we used a mixed effects linear model to analyze the AOP measurements using the two types of cuffs in males and females. Separate analyses were performed for the arms and legs. A mixed effects linear model was also used to compare vessel diameter and blood flow data between males and females and between the arm and leg within males and females. In addition to the main effects, we also evaluated any interactions.

To determine which independent variables were predictive of AOP; age, sex, SBP, DBP arm circumference, arm volume, skinfold thickness, vessel diameter and blood flow were used in a step‐forward regression model to estimate AOP. How well the model fit the data was determined using the Akaike Information Criterion (AIC). The analysis was performed separately for brachial artery AOP and superficial femoral artery AOP.

## 3 RESULTS

Male participants were significantly (*p* < 0.05) taller, heavier, and had higher SBP than the female participants (Table 1). There were no significant sex differences in age, BMI, or resting DBP and MAP.

**TABLE 1:** Participant characteristics.

	Males (*N* = 21)	Females (*N* = 21)	Difference
Age (years)	26.4 ± 2.7	25.9 ± 3.7	3.6 ± 1.0
Height (cm)	179.8 ± 7.5	165.8 ± 6.1	14.0 ± 2.1*
Body mass (kg)	80.9 ± 15.9	68.4 ± 15.7	12.6 ± 4.8*
BMI (kg/m^2^)	24.9 ± 4.0	24.8 ± 4.9	0.14 ± 1.4
SBP (mmHg)	128.8 ± 13.7	115.3 ± 10.2	13.5 ± 3.7*
DBP (mmHg)	82.1 ± 9.1	81.8 ± 9.8	0.24 ± 2.93
MAP (mmHg)	97.5 ± 9.9	92.9 ± 9.5	4.62 ± 3.01

*Note*: Values are mean ± SD.

Abbreviations: BMI, body mass index; DBP, diastolic blood pressure; MAP, mean arterial pressure; SBP, systolic blood pressure.

* Significant difference between males and females (*p*‐value < Bonferroni‐adjusted value of 0.007).

There were no sex differences in arm or leg circumferences or volumes (Table 2). Skinfold thicknesses of the upper arm and thigh were significantly greater in females than in males (*p* < 0.05) (Table 2).

**TABLE 2:** Limb characteristics.

	Males (*N* = 21)	Females (*N* = 21)	Difference
Skinfold (mm)			
Arm	5.45 ± 2.2	10.1 ± 7.4	−4.6 ± 1.6*
Leg	13.7 ± 5.8	27.4 ± 9.6	−13.7 ± 2.4*
Circumference (cm)			
Arm	29.3 ± 4.1	27.2 ± 3.9	2.09 ± 1.23
Leg	52.9 ± 5.6	51.2 ± 4.9	1.79 ± 1.63
Volume (m^3^)			
Arm	0.03 ± 0.01	0.03 ± 0.01	−0.002 ± 0.003
Leg	0.16 ± 0.04	0.15 ± 0.04	−0.01 ± 0.013

*Note*: Values are mean ± SD.

* Significant difference between males and females (*p*‐value < Bonferroni‐adjusted value of 0.008).

**FIGURE 1:**
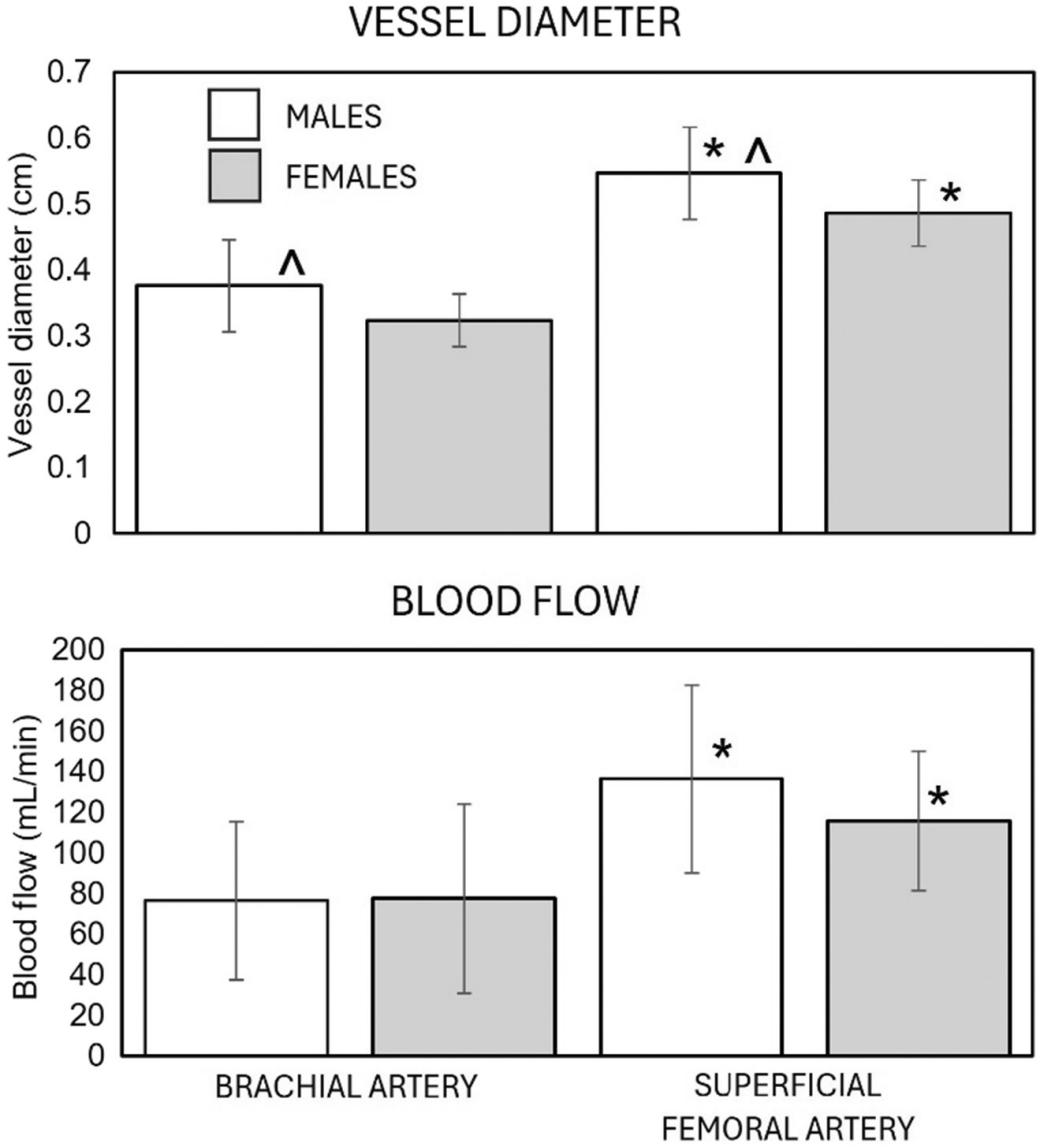
Differences in vessel diameter and blood flow. *Vessel diameter and blood flow are significantly greater in the superficial femoral artery than the brachial artery. ^Superficial femoral and brachial arteries are significantly larger in males than in females.

Differences in vessel diameter and blood flow between males and females and between the brachial and superficial femoral arteries are illustrated in Figure 1. For vessel diameter, there were significant main effects for gender and limb (arm vs. leg). The brachial artery and the superficial femoral artery were significantly larger in males than in females (*p* = 0.002). In both males and females, the superficial femoral artery was significantly (*p* < 0.001) larger than in the brachial artery. For blood flow, there was a significant (*p* < 0.001) main effect for limb. In both males and females, blood flow (mL/min) was significantly (*p* = 0.001) greater in the superficial femoral artery than in the brachial artery. Although there was a significant gender‐by‐limb interaction (*p* = 0.049), post hoc analysis showed that there were no sex differences in blood flow (mL/min) in the brachial artery (*p* = 0.939) and the superficial femoral artery (*p* = 0.121).

**FIGURE 2:**
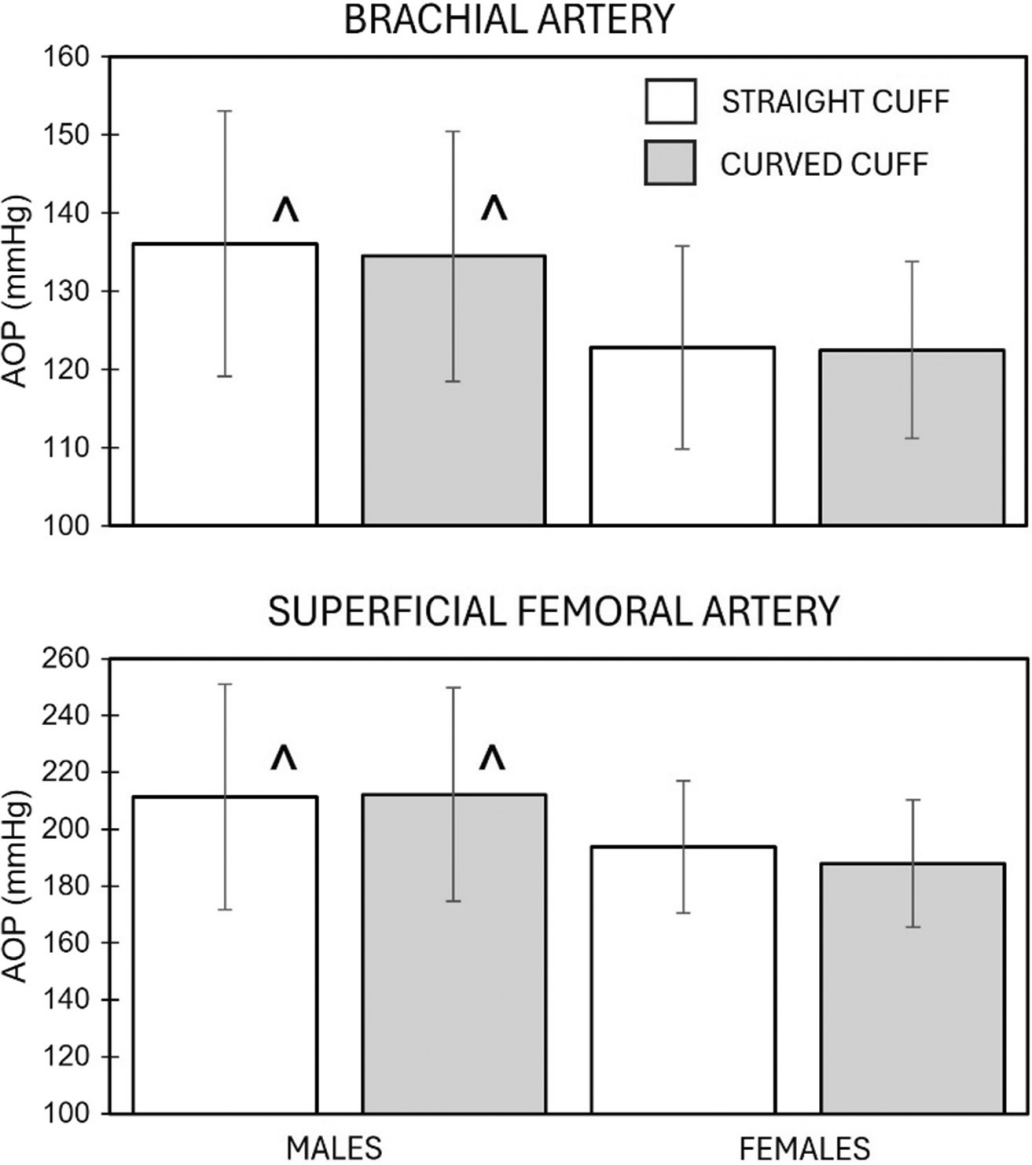
Differences in arterial occlusion pressure. There were no significant differences in arterial occlusion pressure (AOP) when measured using straight or curved cuffs in either the brachial or superficial femoral arteries in males or females. ^The AOP was greater in males than in females in both the brachial and superficial femoral arteries when using a straight and curved cuff to measure AOP.

Overall (combined males/females, combined arm/leg, *n* = 84) the difference in AOP (1.7 ± 10.2 mmHg) measured with a straight (166.0 ± 45.1 mmHg) versus curved (164.3 ± 44.1 mmHg) cuff was not significant (*p* = 0.311). The overall (combined males/females; *n* = 42) differences in AOP measured with a straight and curved cuff in the superficial femoral artery (202.6 ± 33.3, 200.1 ± 32.8 mmHg; *p* = 0.363) and brachial artery (129.5 ± 16.4, 128.5 ± 15.1 mmHg; *p* = 0.427), respectively, were not significant. Differences in AOP of the brachial and superficial femoral arteries between and within males and females using straight and curved blood flow restriction cuffs are reported in Figure 2.

The main effect for type of cuff was not significant when measuring the AOP of the brachial (*p* = 0.346) or superficial femoral (*p* = 0.208) arteries. There were no significant differences in AOP measured in the brachial or superficial femoral arteries when using a straight or curved cuff in males or females (Figure 2). There was a significant main effect for gender in the brachial (*p* = 0.007) and superficial femoral (*p* = 0.035) arteries. The AOP of the brachial and superficial femoral arteries was greater in males than in females regardless of the cuff used to measure AOP. The correlations between measures of AOP when using straight and curved cuffs in the arms and legs in males and females ranged from 0.842 and 0.947.

Based on the regression analysis of AOP data, the best‐fit model (lowest AIC) that predicted brachial artery AOP included SBP and limb volume as independent variables, together accounting for 74% (multiple *R* ^2^ = 0.742) of the variance in AOP. After fitting the model with these two variables, all other variables were not significant. The best‐fit model that predicted superficial femoral artery AOP included SBP, DBP, thigh circumference, and vessel diameter, together accounting for 74% (multiple *R* ^2^ = 0.742) of the variance in AOP. All other variables were not significant.

## 4 DISCUSSION

This paper adds to our current understanding of measuring AOP in that we report no significant differences in AOP when measured using straight and curved blood flow restriction cuffs in the arm or thigh in males and females. We also report sex differences in AOP in the arm and the thigh when using a straight and curved cuff. Perhaps for the first time in the context of BFR training, we report differences in vessel size and blood flow between the brachial and superficial femoral arteries and sex differences in vessel size of both arteries. We also demonstrate that vessel diameter is predictive of AOP of the superficial femoral artery.

### 4.1 Differences in arterial occlusion pressure when using straight and curved cuffs

The straight and curved cuffs used in this study were the same size and made of the same material. Therefore, it was anticipated that the AOP measured with both cuffs would be similar in the arm and the leg. The results of this study indicate that the differences in AOP when using straight and curved cuffs were not significant (Figure 2). The average difference in AOP of the brachial and superficial femoral artery when using the two cuffs was less than 2 and 6 mmHg, respectively. The results of this study are contrary to those previously reported in 1993 (Pedowitz et al., 1993). Pedowitz et al. (1993) reported that compared to using a straight cuff, measures of AOP using a curved cuff were on average 25.4 mmHg lower in the thigh and 4.3 mmHg lower in the arm. Differences between this study and that of Pedowitz et al. (1993) may account for the disparity in the data. For example, we used nylon cuffs that were 10 cm wide, whereas Pedowitz et al. used 8 cm wide cuffs of undisclosed material. Pedowitz et al. inflated the blood flow restriction cuff with a Kidde tourniquet inflator (W. Kidde, Bloomfield, NJ, USA) connected to a pressure transducer, whereas we inflated the cuff using a hand‐held sphygmomanometer. Pedowitz et al. used a Doppler that produced an auditory signal to detect blood flow and measure AOP, whereas we used Doppler ultrasound and the absence of color flow and pulse waves to determine AOP.

Although the differences in AOP measured using a straight and narrow cuff in this study were small and not statistically significant, the differences in AOP tended to be larger when measuring AOP of the superficial femoral artery in the thigh compared to the brachial artery at the upper arm. This could be due to the greater tapering of the thigh compared to the upper arm. We suspect that differences in AOP when measured using straight and curved cuffs may be greater in limbs that have a greater tapering. When measuring AOP on a more tapered limb, a straight cuff may apply asymmetric pressures to the tissue beneath the cuff, thereby resulting in a higher AOP (Rolnick et al., 2023). This would support the data previously reported by Pedowitz et al. (1993). Nevertheless, in this study, the AOP measured using a straight cuff was greater than the AOP measured using a curved cuff in the arms of 24 subjects (58%) and the legs of 23 subjects (56%). As we are unaware of other well‐designed studies comparing AOP measured using straight and curved cuffs, further research using straight and curved cuffs is warranted.

Although the average difference in AOP when using the two cuffs reported in this study is small and not statistically significant, this data should be interpreted with some caution. For example, when measuring AOP of the brachial artery, there was a 30 mmHg range (−16 to +14 mmHg) in the difference in AOP measured using the two cuffs. When measuring AOP of the superficial femoral artery, the range was greater (44 mmHg; −18 to +26 mmHg). Considering the recommendation to use a restrictive pressure representing 40%–80% of the AOP during BFR training (Cognetti et al., 2022; Hughes et al., 2017; McEwen et al., 2019; Patterson et al., 2019; Scott et al., 2015), a difference of 26 mmHg in AOP between the two cuffs would result in cuff pressure used during BFR training that is 10–20 mmHg higher than intended. The use of excessive restrictive cuff pressures could increase discomfort and reduce tolerance to BFR training (Nascimento et al., 2020; Spitz et al., 2022).

### 4.2 Sex differences in arterial occlusion pressure

When combining AOP data from both limbs (arms and legs) and both cuffs (straight and curved), AOP was significantly greater in males (173.5 ± 48.2 mmHg) than in females (156.7 ± 38.8). Post hoc testing indicated that significant sex differences in the AOP of the brachial artery and superficial femoral artery were consistent when using the straight and curved cuffs (Figure 2). The results of this study concur with those of previous studies reporting sex differences in AOP of the brachial artery (Jessee et al., 2016; Mouser et al., 2017; Vehrs et al., 2024) and superficial femoral artery (Tafuna'i et al., 2021). A key determinant of AOP is the circumference of the limb (Mattocks et al., 2018; McEwen et al., 2019; Scott et al., 2015; Tafuna'i et al., 2021). Thus, differences in limb circumference could explain differences in AOP between males and females. In this study, sex differences in AOP were evident even though there were no significant sex differences in circumference or volume of the upper arm or thigh (Table 2). Other factors, such as the significant sex differences in arm and thigh skinfold thicknesses (Table 2) and the diameter of the brachial and superficial femoral arteries (Figure 1) may have contributed to the sex differences in AOP. We are unaware of previous studies evaluating methods of measuring AOP that have also evaluated the differences in vessel size or blood flow. In this study, the independent variables that entered the model to predict brachial artery AOP included SBP and limb volume. Limb volume was previously reported to be a significant independent variable predictive of brachial artery AOP, accounting for 43% of the variance in AOP (Vehrs et al., 2024). Previous studies have also reported SBP to be a significant independent variable predictive of AOP (Brown et al., 2018; Loenneke et al., 2015). The independent variables that entered the model to predict superficial femoral artery AOP included SBP, DBP, thigh circumference, and vessel diameter. This is perhaps the first study to report sex differences in vessel diameter and the contribution of vessel diameter as an independent predictor of AOP. Mouser et al. (2017) previously reported significant sex differences in brachial artery blood flow and AOP but did not evaluate the influence of blood flow as an independent predictor of AOP. In this study, limb circumference and vessel diameter were significant independent predictors of AOP only in the leg. One explanation for this may be the larger circumference of the leg (compared to the arm) and the larger diameter of the superficial femoral artery (compared to the brachial artery). Because of mounting evidence supporting sex differences in AOP, further investigation of factors that contribute to AOP that may also explain differences in AOP between individuals (including males and females) is warranted.

Factors that are predictive of AOP are inconsistently reported in the literature making it difficult to ascertain the variables that are predictive of AOP. For example, limb circumference; limb volume; skinfold thickness; and SBP, DBP, and MAP have been reported as independent predictors of AOP in some, but not all studies. Thus, it is likely that variables that are predictive of AOP vary between individuals, and/or there are unknown variables that are yet to be identified that are predictive of AOP. Evaluation of variables such as fitness level, training status, and vascular health may lead to a better understanding of the differences in AOP between individuals.

### 4.3 Study limitations

This study is not without limitations. This study included physically active, apparently healthy young adult coeds who did not self‐report a history of or risk factors for cardiometabolic diseases or thromboembolisms. The results of this study may not apply to other subgroups of the population. In this study, the blood flow restriction cuff was inflated using a hand‐held sphygmomanometer with a gauge ranging from 0 to 300 mmHg with a ±3 mmHg certified accuracy and a continuous cuff inflation protocol. Other methods to inflate the cuff may result in different results. The blood flow restriction cuffs used in this study were of the same size and made of the same material (nylon). Blood flow restriction cuffs made of other types of material may yield different results. Investigators in this study were well trained in the use of the US and the measurement of AOP. Less‐experienced individuals may achieve different results.

## 5 CONCLUSIONS

Both brachial and superficial femoral artery AOP can be measured with straight or curved cuffs. The choice of cuff may depend on the taper of the limb, the fit of the cuff on the limb, and how well the cuff remains in place during BFR training. Sex differences in AOP of arm and leg continue to require additional investigation. As this study is unique in the comparison of blood vessel characteristics (vessel size and blood flow) between the brachial and superficial femoral arteries and between males and females, influence of these variables on AOP needs further investigation. To further evaluate independent variables predictive of AOP, future studies that evaluate methods of measuring AOP should include as many variables as reasonably possible, including sex comparisons of AOP, limb circumference, limb volume, a measure of limb composition, blood pressures, vessel size, and blood flow.

## FUNDING INFORMATION

No funding information provided.

## CONFLICT OF INTEREST STATEMENT

The authors report that there are no conflicts of interest to declare.

## ETHICS STATEMENT

This study was reviewed and approved by the Institutional Review Board (IRB2023‐256) prior to the collection of any data.

## ARTIFICIAL INTELLIGENCE

Artificial Intelligence was not used in the preparation of this manuscript.

## Data Availability

The data that support the findings of this study are available upon reasonable request from the corresponding author.
